# Association between social media influence and postoperative satisfaction following upper eyelid blepharoplasty: a 500-patient study

**DOI:** 10.3389/fsurg.2026.1841059

**Published:** 2026-08-06

**Authors:** Saif Al-dossari

**Affiliations:** Department of Surgery, College of Medicine, King Faisal University, Al-Ahsa, Saudi Arabia

**Keywords:** aesthetic surgery, expectation management, Instagram, patient-reported outcomes, preoperative counseling, TikTok

## Abstract

**Purpose:**

To examine whether the degree of social media influence on decision-making is associated with postoperative satisfaction among adults undergoing upper eyelid blepharoplasty.

**Patients and methods:**

A quantitative observational comparative cross-sectional study was conducted among 500 adults who underwent elective upper eyelid blepharoplasty at the polyclinics of King Faisal University, Saudi Arabia, and attended the 3-month follow-up. Participants self-reported the extent to which social media influenced their decision (strong, moderate, minimal, or none) using a structured questionnaire. Social media platforms and content types were recorded, and postoperative satisfaction at three months was assessed using a structured satisfaction tool. Descriptive statistics summarized patterns of influence and satisfaction, and satisfaction rates were compared across influence categories (*p* < 0.05).

**Results:**

Overall, 440/500 (88%) participants reported some degree of social media influence: 220/500 (44%) strong, 140/500 (28%) moderate, and 80/500 (16%) minimal; 60/500 (12%) reported no influence. Instagram and TikTok were the most frequently cited platforms (41% and 27%, respectively), and before-and-after images were the most influential content type (64%). Postoperative satisfaction at three months was high overall but differed by influence intensity: 86% in the strong influence group (190/220), 96% in the moderate influence group (135/140), and 93% among patients with minimal or no influence (130/140). Among dissatisfied strongly influenced patients (*n* = 30), the most commonly reported reasons were more visible scarring than expected (50%) and longer-than-expected swelling (27%).

**Conclusion:**

Most patients reported social media influence when pursuing upper eyelid blepharoplasty. Although satisfaction was high overall, strong social media influence was associated with slightly lower satisfaction at three months, largely related to recovery expectations rather than major aesthetic failure. Incorporating targeted counseling about realistic outcomes, scar maturation, and recovery timelines may help align expectations and optimize patient satisfaction.

## Introduction

Upper eyelid blepharoplasty is among the most commonly performed aesthetic surgical procedures worldwide, offering both functional and cosmetic benefits through the correction of dermatochalasis, ptosis, and periorbital aging changes (1). High postoperative satisfaction has traditionally been attributed to the procedure's predictability, relatively low complication rates, and visible aesthetic improvement (2). However, patient satisfaction in cosmetic surgery is not determined solely by technical outcomes; it is strongly influenced by preoperative expectations, psychosocial factors, and individual perceptions of aesthetic success (3). In recent years, the rapid expansion of social media platforms has introduced a new and powerful determinant of patient expectations, fundamentally reshaping how individuals perceive beauty, surgical outcomes, and success in aesthetic medicine (4).

Social media platforms such as Instagram, TikTok, and YouTube have become primary sources of health-related information, particularly in aesthetic and cosmetic medicine. These platforms provide continuous exposure to curated before-and-after images, patient testimonials, influencer narratives, and short-form educational videos produced by surgeons and non-medical content creators alike (5). While such content may enhance awareness and accessibility of aesthetic procedures, it often presents idealized or selectively edited outcomes, compresses recovery timelines, and underrepresents postoperative variability and complications (6). Consequently, patients increasingly arrive at consultations with highly specific visual expectations that may not align with anatomical limitations or realistic surgical outcomes (7).

Expectation formation is a critical determinant of postoperative satisfaction in cosmetic surgery. Previous research has demonstrated that unrealistic or overly idealized expectations are associated with increased dissatisfaction, even in cases where objective surgical outcomes are clinically successful (8, 9). Unlike reconstructive procedures, aesthetic surgery outcomes are judged largely through subjective self-assessment, making patients particularly vulnerable to dissatisfaction when perceived results fail to match preoperative mental images (10). Social media, by emphasizing aesthetic perfection and uniformity, may amplify this expectation gap, especially when patients seek to replicate the appearance of celebrities or influencers whose images may be filtered, staged, or digitally altered (11).

Several studies have explored the influence of social media on the decision to pursue cosmetic surgery, reporting that online exposure significantly increases interest in aesthetic procedures, particularly among younger and middle-aged adults (12). However, far fewer investigations have examined how the *degree* of social media influence affects postoperative satisfaction once surgery has been performed (13). Existing literature suggests mixed findings: while some studies report no significant reduction in satisfaction, others indicate that patients heavily influenced by social media may experience disappointment related to scarring, swelling duration, or subtle asymmetries that deviate from idealized online portrayals (14). Importantly, many prior studies are limited by small sample sizes, heterogeneous procedures, or lack of stratification by influence intensity.

Upper eyelid blepharoplasty represents a particularly relevant procedure for examining this relationship. Outcomes are highly visible, frequently compared against online images, and sensitive to minor variations that may be imperceptible clinically yet meaningful to patients (15). Additionally, recovery-related factors such as edema resolution, scar maturation, and eyelid symmetry evolve over time, often contrasting sharply with the immediate “after” images commonly depicted on social media (16). Understanding how social media influence shapes satisfaction in this context has direct implications for preoperative counseling, informed consent, and expectation management (17).

Therefore, the present study aims to examine the association between social media influence and postoperative satisfaction following upper eyelid blepharoplasty in a large cohort of 500 patients. By stratifying patients according to the degree of social media influence and comparing postoperative satisfaction rates, this study seeks to clarify whether strong social media exposure is associated with reduced satisfaction despite overall favorable surgical outcomes. Identifying this relationship is essential for guiding surgeons in tailoring preoperative discussions, mitigating unrealistic expectations, and optimizing patient-centered care in the digital era of aesthetic surgery.

## Material and methods

### Study design

This study employed a quantitative observational comparative cross-sectional design to examine the association between the degree of social media influence and postoperative satisfaction among patients undergoing upper eyelid blepharoplasty. A comparative approach was adopted to allow stratification of participants based on self-reported levels of social media influence and to evaluate differences in postoperative satisfaction outcomes across influence categories.

### Study setting

The study was conducted at the **Polyclinics of King Faisal University (KFU), Al-Ahsa, Saudi Arabia**, a tertiary academic healthcare setting that provides outpatient surgical and aesthetic services to a diverse adult population. Upper eyelid blepharoplasty procedures at the polyclinics are performed by board-certified surgeons following standardized clinical protocols, ensuring consistency in surgical technique, perioperative care, and follow-up assessment.

### Sample and sampling

The study included 500 adult patients who underwent elective upper eyelid blepharoplasty at KFU polyclinics from June 2025 to December 2025 during the study period. A consecutive sampling technique was used, whereby all eligible patients who met the inclusion criteria and attended their postoperative follow-up visit were invited to participate until the target sample size was achieved.

Inclusion criteria were: age ≥30 years, completion of primary upper eyelid blepharoplasty, and attendance at the 3-month postoperative follow-up visit. Exclusion criteria included revision blepharoplasty, combined major facial procedures, documented psychiatric illness affecting body image perception, or incomplete postoperative data. Consecutive sampling minimized selection bias and enhanced the representativeness of real-world clinical practice within the academic polyclinic setting.

### Data collection tools

Data were collected using three structured instruments, administered in Arabic.

The Sociodemographic and Clinical Data Form was developed by the research team to collect baseline patient information, including age, sex, educational level, prior cosmetic procedures, and clinical indications for surgery. This form ensured contextual understanding of participant characteristics and allowed adjustment for potential confounding variables in the analysis.

The Social Media Influence on Surgical Decision Questionnaire was a structured self-report tool developed for this study based on existing literature on digital health influence and cosmetic decision-making. The tool aimed to assess the degree and nature of social media influence on the patient's decision to undergo blepharoplasty. It consisted of items evaluating exposure to social media content, primary platforms used, type of content viewed (e.g., before-and-after images, testimonials, influencer posts), and perceived impact on decision-making. Responses were scored on a Likert-type scale, and participants were categorized into strong, moderate, minimal, or no social media influence groups based on predefined scoring thresholds. Content validity was established through expert review by three clinicians with experience in aesthetic surgery and patient-reported outcome research. Internal consistency reliability in the current sample was acceptable (Cronbach's *α* > 0.80). The questionnaire was developed originally in English, translated into Arabic using forward–backward translation, and reviewed for linguistic clarity and cultural appropriateness. Content validity was further quantified using the Content Validity Index (CVI): item-level CVI (I-CVI) ranged from 0.80 to 1.00, and the scale-level CVI (S-CVI/Ave) was 0.91, indicating strong content validity. Pilot testing was conducted with 20 patients (not included in the final sample) to assess item clarity, response format comprehension, and time to completion; minor wording adjustments were made based on pilot feedback. Cross-cultural adaptation followed standard international guidelines, including forward translation into Arabic by two bilingual translators, independent backward translation into English by two additional translators, expert committee review for semantic and conceptual equivalence, and cognitive debriefing with target-population members to ensure cultural appropriateness. Construct validity was not formally evaluated through exploratory or confirmatory factor analysis in the present study; this is acknowledged as a methodological limitation (see Limitations).

Postoperative satisfaction was measured using the Postoperative Satisfaction Assessment Tool, adapted from validated cosmetic surgery satisfaction frameworks. The tool aimed to assess patients' subjective evaluation of surgical outcomes at three months postoperatively, covering aesthetic satisfaction, perceived naturalness of results, and alignment between expectations and outcomes. Items were rated on a Likert scale, with higher scores indicating greater satisfaction. The instrument demonstrated strong internal consistency in the present study (Cronbach's *α* > 0.85). The Arabic version underwent forward–backward translation, expert validation, and pilot testing to ensure semantic equivalence and cultural relevance.Item-level CVI (I-CVI) for this instrument ranged from 0.83 to 1.00, with a scale-level CVI (S-CVI/Ave) of 0.93.

Both instruments are provided in full as Supplementary Material (Supplementary File S1: Social Media Influence on Surgical Decision Questionnaire; Supplementary File S2: Postoperative Satisfaction Assessment Tool) to enhance transparency and reproducibility.

### Data collection procedure

Eligible patients were identified during routine postoperative follow-up visits at three months after surgery. After providing verbal and written study information, informed consent was obtained. Data collection was conducted in a private clinical setting to ensure confidentiality. Questionnaires were self-administered, with assistance provided when necessary to minimize response bias. All data were anonymized and coded prior to analysis.

### Data analysis

Data were analyzed using IBM SPSS Statistics, version 29.0 (IBM Corp., Armonk, NY, USA). Descriptive statistics (frequencies, percentages, means, and standard deviations) were used to summarize participant characteristics and study variables. Comparative analyses were conducted to examine differences in postoperative satisfaction across social media influence categories using appropriate inferential tests. Statistical significance was set at *p* < 0.05. Findings were reported following STROBE recommendations for observational studies.

### Ethical considerations

Ethical approval was obtained from the Institutional Review Board of King Faisal University. Participation was voluntary, and informed consent was obtained from all participants. Confidentiality was maintained by anonymizing all data and restricting access to the research team only. The study was conducted in accordance with the principles of the Declaration of Helsinki and applicable institutional ethical guidelines.

## Results

Table 1 presents the distribution of patients according to the degree of social media influence on their decision to undergo upper eyelid blepharoplasty among the 500 participants included in the study. Nearly half of the patients (220/500, 44%) reported a strong influence, indicating that social media content served as a direct source of inspiration for pursuing the procedure. A further 28% (140/500) described a moderate level of influence, suggesting that exposure to social media contributed to, but was not solely responsible for, their decision-making process. In contrast, 16% (80/500) indicated minimal influence, reflecting passive exposure without a decisive role, while 12% (60/500) reported no social media influence at all. Overall, the table demonstrates that the majority of patients (440/500) experienced some level of social media influence, with influence intensity varying across the cohort.

**Table 1:** Degree of social media influence on the decision to undergo upper eyelid blepharoplasty (*n* = 500).

Influence level	n/N	%
Strong influence (direct inspiration)	220/500	44
Moderate influence (some impact)	140/500	28
Minimal influence (seen but not decisive)	80/500	16
No influence	60/500	12

Table 2 summarizes the social media platforms most frequently cited by patients who reported some degree of social media influence on their decision to undergo upper eyelid blepharoplasty (*n* = 440). Instagram emerged as the most commonly reported platform, cited by 180 of the influenced patients (41%), followed by TikTok, which was reported by 120 patients (27%). YouTube was identified by 80 patients (18%), while Facebook and Snapchat were less frequently cited, accounting for 40 patients (9%) and 20 patients (5%), respectively.

**Table 2:** Social media platforms most frequently cited by influenced patients (*n* = 440).

Platform	n/N	%
Instagram	180/440	41
TikTok	120/440	27
YouTube	80/440	18
Facebook	40/440	9
Snapchat	20/440	5

Table 3 outlines the types of social media content that influenced patients' decisions to undergo upper eyelid blepharoplasty among the 440 participants who reported social media exposure. Before-and-after images were the most frequently cited content type, reported by 280 patients (64%), indicating that visual outcome comparisons were the dominant form of influence. Patient testimonials were identified by 90 patients (20%), reflecting the role of shared personal experiences in shaping surgical decisions. Surgeon educational videos were cited by 50 patients (11%), suggesting a more limited but notable influence of informational content. Celebrity or influencer-related surgical content was the least frequently reported, cited by 20 patients (5%).

**Table 3:** Types of social media content influencing the decision to undergo surgery (*n* = 440).

Content type	n/N	%
Before-and-after images	280/440	64
Patient testimonials	90/440	20
Surgeon educational videos	50/440	11
Celebrity or influencer surgery	20/440	5

Table 4 presents the distribution of age groups among patients who reported social media influence on their decision to undergo upper eyelid blepharoplasty (*n* = 440). The largest proportion of influenced patients fell within the 30–39-year age group, accounting for 180 individuals (41%), followed by those aged 40–49 years, who comprised 150 patients (34%). Patients aged 50–59 years represented 80 individuals (18%), while those aged 60–75 years accounted for a smaller proportion, with 30 patients (7%).

**Table 4:** Age groups most affected by social media influence (*n* = 440).

Age group (years)	n/N	%
30–39	180/440	41
40–49	150/440	34
50–59	80/440	18
60–75	30/440	7

Table 5 summarizes the distribution of preoperative expectation levels among patients who reported social media influence on their decision to undergo upper eyelid blepharoplasty (*n* = 440). Half of the influenced patients (220/440, 50%) reported having realistic but detailed expectations regarding surgical outcomes. A substantial proportion, 170 patients (39%), expressed very high expectations, indicating anticipation of near-ideal or optimal results. In contrast, 50 patients (11%) described their expectations as unclear or vague.

**Table 5:** Preoperative expectation levels in social media–influenced patients (*n* = 440).

Expectation level	n/N	%
Very high expectations	170/440	39
Realistic but detailed	220/440	50
Unclear or vague	50/440	11

Table 6 presents postoperative satisfaction outcomes at three months following upper eyelid blepharoplasty according to the level of social media influence among the 500 patients included in the study. Patients reporting moderate social media influence demonstrated the highest satisfaction rate, with 135 of 140 patients satisfied (96%), and a relatively small number reporting dissatisfaction. Patients with minimal or no social media influence also showed high satisfaction, with 130 of 140 patients satisfied (93%). In comparison, patients reporting strong social media influence had a lower satisfaction rate, with 190 of 220 patients satisfied (86%) and a higher proportion reporting dissatisfaction.

**Table 6:** Postoperative satisfaction at three months according to social media influence level (*n* = 500).

Influence level	Satisfied n/N	Dissatisfied n/N	Satisfaction %
Strong influence	190/220	30/220	86
Moderate influence	135/140	5/140	96
Minimal or no influence	130/140	10/140	93

Table 7 describes the reported sources of postoperative disappointment among patients who were strongly influenced by social media and expressed dissatisfaction following upper eyelid blepharoplasty (*n* = 30). The most frequently reported source of disappointment was scarring that appeared more visible than expected, cited by 15 patients (50%). Prolonged postoperative swelling was reported by 8 patients (27%), indicating concerns related to the duration of recovery rather than the final outcome. Minor asymmetry when compared with online images was reported by 5 patients (17%), while dissatisfaction related to the result not being identical to an influencer or online reference image was reported by 2 patients (6%).

**Table 7:** Reported sources of postoperative disappointment among strongly influenced patients (*n* = 30).

Source of disappointment	n/N	%
Scarring more visible than expected	15/30	50
Swelling lasted longer than expected	8/30	27
Minor asymmetry compared with online images	5/30	17
Result not identical to influencer	2/30	6

## Discussion

The present study examined the association between social media influence and postoperative satisfaction following upper eyelid blepharoplasty in a large cohort of patients attending King Faisal University polyclinics in Saudi Arabia. The findings demonstrate that social media plays a substantial role in motivating patients to pursue aesthetic surgery, with the majority of participants reporting some level of influence. Importantly, although overall postoperative satisfaction remained high across all groups, patients reporting strong social media influence exhibited comparatively lower satisfaction at three months postoperatively (18). From a psychological perspective, these findings highlight the critical role of expectation formation, cognitive comparison processes, and digitally mediated social norms in shaping postoperative evaluations (19).

Consistent with prior research, visually oriented platforms such as Instagram and TikTok emerged as the dominant sources of influence, and before-and-after images were the most impactful content type (20). Psychological theories of social comparison provide a useful framework for interpreting this pattern. According to social comparison theory, individuals evaluate themselves and their outcomes by comparing them to salient reference standards, particularly when objective criteria are ambiguous (21). In aesthetic surgery, where outcomes are largely subjective and appearance-based, curated online images may serve as powerful upward comparison targets, increasing perceived discrepancy between expected and actual results (22).

The graded relationship observed between social media influence and postoperative satisfaction is particularly noteworthy. Patients with moderate social media influence demonstrated the highest satisfaction, whereas those with strong influence reported lower satisfaction despite undergoing the same procedure within the same clinical setting (23). This finding suggests that social media exposure is not inherently detrimental; rather, its psychological impact may depend on intensity and interpretive framing (24). Moderate exposure may enhance procedural understanding, normalize recovery experiences, and support informed decision-making, whereas strong exposure may foster rigid or idealized outcome expectations that are difficult to reconcile with individualized surgical results (25).

Preoperative expectation patterns observed in this study further support this interpretation. Nearly two-fifths of socially influenced patients reported very high expectations, while half reported realistic but highly detailed expectations (26). Expectation theory posits that satisfaction is largely determined by the congruence between anticipated and actual outcomes rather than objective outcome quality alone (27). When expectations are unrealistically elevated, even successful outcomes may be perceived as insufficient. This phenomenon has been consistently documented in cosmetic surgery, dermatology, and other elective medical contexts (28, 29).

The sources of postoperative dissatisfaction identified among strongly influenced patients were predominantly related to recovery characteristics, such as scar visibility and prolonged swelling, rather than major aesthetic failure (30). From a psychological standpoint, this finding underscores the role of temporal distortion in social media content. Online representations frequently depict immediate or short-term “after” results, often omitting the normal trajectory of healing, scar maturation, and edema resolution (31). Patients who internalize these representations may misinterpret normal postoperative changes as deviations or complications, leading to dissatisfaction despite favorable long-term prognosis (32).

Age-related patterns observed in this study also merit consideration. Social media influence was most prominent among patients aged 30–49 years, aligning with demographic trends in platform engagement and digital literacy (33). However, the presence of social media influence across all age groups suggests that digital aesthetic norms are not confined to younger populations. From a lifespan psychology perspective, middle-aged adults may be particularly susceptible due to heightened salience of aging-related appearance changes combined with frequent exposure to rejuvenation-focused content (34).

Cultural context is also relevant when interpreting these findings. In Saudi Arabia, social media usage is among the highest globally, and aesthetic procedures have become increasingly normalized within broader sociocultural narratives of self-care and professional presentation (12). The intersection of collectivist cultural values, visual self-presentation, and rapid digital dissemination may amplify normative pressures toward aesthetic enhancement, while simultaneously shaping expectations of outcome perfection. Understanding these cultural dynamics is essential for contextualizing patient psychology and tailoring preoperative counseling strategies.

### Implications of the study

The findings of this study have important clinical, psychological, and practice-based implications for aesthetic surgery in the digital era. First, the high prevalence of social media influence among patients undergoing upper eyelid blepharoplasty highlights the need for clinicians to routinely assess patients' digital exposure as part of the preoperative consultation. Understanding the degree and nature of social media influence can help surgeons identify patients who may hold elevated or idealized expectations and therefore require more detailed counseling.

Second, the observation that patients with moderate social media influence demonstrated the highest postoperative satisfaction suggests that social media is not inherently detrimental. When appropriately contextualized, online content may enhance procedural awareness, normalize interest in aesthetic surgery, and support informed decision-making. This underscores the importance of guiding patients toward educational, surgeon-generated content and away from unrealistic or heavily curated imagery.

Third, the findings emphasize the psychological role of expectation management in optimizing patient-reported outcomes. Structured discussions addressing common misconceptions derived from before-and-after images—such as recovery timelines, scar maturation, and individual anatomical variability—may reduce postoperative disappointment. Incorporating expectation-focused counseling strategies into routine practice may improve satisfaction without altering surgical technique.

Finally, at an institutional level, the results support the development of standardized preoperative educational materials that directly address social media influence. Such interventions may enhance patient-centered care, improve satisfaction metrics, and reduce dissatisfaction driven by expectation–outcome mismatch rather than surgical quality.

### Limitations of the study

Several limitations should be considered when interpreting the findings of this study. First, the cross-sectional design limits the ability to establish causal relationships between social media influence and postoperative satisfaction. Although associations were identified, longitudinal assessment would be required to determine temporal and causal pathways.

Second, social media influence and satisfaction were assessed using self-reported measures, which may be subject to recall bias or social desirability bias. Patients may underreport dissatisfaction or overestimate the role of social media depending on personal perceptions or expectations.

Third, satisfaction was evaluated at three months postoperatively, a period during which some aspects of healing, particularly scar maturation, may still be ongoing. Longer follow-up periods could provide additional insight into whether satisfaction levels change over time as surgical outcomes stabilize.

Fourth, the study was conducted at a single academic polyclinic in Saudi Arabia, which may limit generalizability to other cultural contexts, healthcare systems, or private practice settings. Cultural norms related to social media use and aesthetic ideals may influence patient perceptions differently across regions.

Finally, psychological constructs such as body image dissatisfaction, appearance anxiety, or baseline mental health status were not formally assessed, which may have provided deeper insight into individual differences in satisfaction. These unmeasured psychological variables represent potential residual confounders: patients with higher appearance anxiety or body image dissatisfaction may be more susceptible to social media influence and simultaneously more prone to postoperative dissatisfaction, independent of the actual surgical outcome. Future studies should incorporate validated psychological instruments — such as the Body Image Scale, the Social Appearance Anxiety Scale, or the Patient Health Questionnaire — to disentangle the independent contributions of psychological predisposition and social media exposure on postoperative satisfaction.

Sixth, the study-specific questionnaire was not subjected to exploratory or confirmatory factor analysis, and formal construct validity was therefore not established. Although content validity and internal consistency were satisfactory, the absence of factor-analytic evidence limits confirmation of the instrument's latent structure. Future validation studies incorporating structural equation modeling or Rasch analysis would strengthen its psychometric foundation.

## Conclusion

This study demonstrates that social media plays a substantial role in influencing patients' decisions to undergo upper eyelid blepharoplasty, with the majority of patients reporting some degree of digital influence. While overall postoperative satisfaction was high, patients who reported strong social media influence exhibited comparatively lower satisfaction at three months following surgery.

The findings suggest that reduced satisfaction among strongly influenced patients is primarily related to elevated preoperative expectations and comparison with idealized online representations rather than objective surgical shortcomings. Moderate exposure to social media, in contrast, was associated with the highest satisfaction levels.

These results highlight the importance of integrating psychologically informed expectation management and discussion of social media influence into preoperative counseling. Addressing digital expectations proactively may enhance patient satisfaction, improve surgical experiences, and support patient-centered care in contemporary aesthetic practice.

## Data Availability

The datasets presented in this study can be found in online repositories. The names of the repository/repositories and accession number(s) can be found in the article/Supplementary Material.
