# Supplementary material for: Association between social media influence and postoperative satisfaction following upper eyelid blepharoplasty: a 500-patient study
Source: Front Surg. 2026 Aug 6;13:1841059. doi: 10.3389/fsurg.2026.1841059 (PMC13490094; doi:10.3389/fsurg.2026.1841059)
Supplement: Supplementary file 1 [file Table1.docx]

**Supplementary File S1**

**Social Media Influence on Surgical Decision Questionnaire**

*Study: Association Between Social Media Influence and Postoperative Satisfaction Following Upper Eyelid Blepharoplasty: A 500-Patient Study*

Setting: Polyclinics of King Faisal University, Al-Ahsa, Saudi Arabia

Instructions: Please answer each question honestly. There are no right or wrong answers. All responses are anonymous and confidential.

# Section A: Sociodemographic Information

| **Item** | **Response Options** |
| --- | --- |
| A1. Age | Open field (years): ________ |
| A2. Sex | ☐ Male ☐ Female |
| A3. Educational level | ☐ Primary ☐ Secondary ☐ Diploma ☐ Bachelor's ☐ Postgraduate |
| A4. Occupation | Open field: ________ |
| A5. Prior cosmetic procedure(s) | ☐ None ☐ Yes (please specify): ________ |

# Section B: Social Media Usage

| **Item** | **Response Options** |
| --- | --- |
| B1. Do you use social media? | ☐ Yes ☐ No (If No, skip to Section D) |
| B2. How many hours per day do you spend on social media? | ☐ <1 hour ☐ 1–2 hours ☐ 3–4 hours ☐ >4 hours |
| B3. Which platform(s) do you use most? (select all that apply) | ☐ Instagram ☐ TikTok ☐ YouTube ☐ Facebook ☐ Snapchat ☐ Other: ________ |
| B4. Have you ever seen content related to eyelid or cosmetic surgery on social media? | ☐ Yes ☐ No |
| B5. What type of content influenced you most? (select all that apply) | ☐ Before-and-after images ☐ Patient testimonials ☐ Surgeon educational videos ☐ Celebrity/influencer content ☐ Other: ________ |

# Section C: Influence on Surgical Decision

*Rated on a 5-point Likert scale: 1 = Strongly disagree, 2 = Disagree, 3 = Neutral, 4 = Agree, 5 = Strongly agree*

| **Item** | **1 – 2 – 3 – 4 – 5** |
| --- | --- |
| C1. Social media content motivated me to consider eyelid surgery. | ☐ ☐ ☐ ☐ ☐ |
| C2. Before-and-after images I saw online influenced my expectations of the result. | ☐ ☐ ☐ ☐ ☐ |
| C3. Online testimonials from other patients influenced my decision. | ☐ ☐ ☐ ☐ ☐ |
| C4. I sought a surgeon based on their social media profile or posts. | ☐ ☐ ☐ ☐ ☐ |
| C5. Content from celebrities or influencers shaped my idea of ideal outcomes. | ☐ ☐ ☐ ☐ ☐ |
| C6. Social media helped me understand what to expect during recovery. | ☐ ☐ ☐ ☐ ☐ |
| C7. I believe the results shown online are realistic and achievable. | ☐ ☐ ☐ ☐ ☐ |
| C8. Social media was a primary factor in my decision to undergo surgery. | ☐ ☐ ☐ ☐ ☐ |

*Scoring: Total score ≥ 32 = Strong influence; 17–31 = Moderate influence; 9–16 = Minimal influence; ≤ 8 = No influence*

# Section D: Overall Influence Classification

| **Item** | **Response** |
| --- | --- |
| D1. Overall, how much did social media influence your decision to undergo upper eyelid blepharoplasty? | ☐ Strong (direct inspiration) ☐ Moderate (contributed to my decision) ☐ Minimal (seen but not decisive) ☐ No influence |

Thank you for completing this questionnaire.
