# Supplementary material for: Association between social media influence and postoperative satisfaction following upper eyelid blepharoplasty: a 500-patient study
Source: Front Surg. 2026 Aug 6;13:1841059. doi: 10.3389/fsurg.2026.1841059 (PMC13490094; doi:10.3389/fsurg.2026.1841059)
Supplement: Supplementary file 2 [file Table2.docx]

**Supplementary File S2**

**Postoperative Satisfaction Assessment Tool**

*Study: Association Between Social Media Influence and Postoperative Satisfaction Following Upper Eyelid Blepharoplasty: A 500-Patient Study*

Setting: Polyclinics of King Faisal University, Al-Ahsa, Saudi Arabia

Administered at: 3 months postoperatively

Instructions: Please answer each question based on how you feel about your surgical results at this time. All responses are confidential.

# Section A: Aesthetic Satisfaction

*Rated on a 5-point Likert scale: 1 = Very dissatisfied, 2 = Dissatisfied, 3 = Neutral, 4 = Satisfied, 5 = Very satisfied*

| **Item** | **1 – 2 – 3 – 4 – 5** |
| --- | --- |
| A1. I am satisfied with the overall appearance of my eyelids after surgery. | ☐ ☐ ☐ ☐ ☐ |
| A2. The result looks natural. | ☐ ☐ ☐ ☐ ☐ |
| A3. The symmetry of my eyelids meets my expectations. | ☐ ☐ ☐ ☐ ☐ |
| A4. The scar appearance is acceptable to me. | ☐ ☐ ☐ ☐ ☐ |
| A5. The swelling has resolved to a satisfactory degree. | ☐ ☐ ☐ ☐ ☐ |

# Section B: Expectation–Outcome Alignment

*Rated on a 5-point Likert scale: 1 = Strongly disagree, 2 = Disagree, 3 = Neutral, 4 = Agree, 5 = Strongly agree*

| **Item** | **1 – 2 – 3 – 4 – 5** |
| --- | --- |
| B1. The result matches what I expected before surgery. | ☐ ☐ ☐ ☐ ☐ |
| B2. The recovery timeline matched what I was told to expect. | ☐ ☐ ☐ ☐ ☐ |
| B3. The result meets the goals I discussed with my surgeon. | ☐ ☐ ☐ ☐ ☐ |
| B4. If I had known the exact result, I would still have chosen to have this surgery. | ☐ ☐ ☐ ☐ ☐ |
| B5. The result matches images I had seen before surgery (social media, internet, etc.). | ☐ ☐ ☐ ☐ ☐ |

# Section C: Overall Satisfaction

| **Item** | **1 – 2 – 3 – 4 – 5** |
| --- | --- |
| C1. Overall, how satisfied are you with the outcome of your surgery? | ☐ ☐ ☐ ☐ ☐ |
| C2. Would you recommend this procedure to a friend or family member? | ☐ Definitely yes ☐ Probably yes ☐ Unsure ☐ Probably not ☐ Definitely not |

# Section D: Sources of Disappointment (if any)

D1. If you are not fully satisfied with your result, what are the main reasons? (select all that apply)

| **Reason** | **Select** |
| --- | --- |
| Scarring more visible than expected | ☐ |
| Swelling lasted longer than expected | ☐ |
| Minor asymmetry compared with online images | ☐ |
| Result does not match influencer/celebrity appearance | ☐ |
| Inadequate improvement in eyelid appearance | ☐ |
| Other (please specify): ________ | ☐ |

*Scoring: Total score (Sections A+B+C) ≥ 38 = Satisfied; < 38 = Dissatisfied. Higher scores indicate greater satisfaction.*

Thank you for completing this questionnaire.
